# Antimicrobial Use in Pig Farms in the Midwestern Region of Minas Gerais, Brazil

**DOI:** 10.3390/antibiotics13050403

**Published:** 2024-04-28

**Authors:** Bruno César de Oliveira, Idael Christiano de Almeida Santa Rosa, Maurício Cabral Dutra, Felipe Norberto Alves Ferreira, Andrea Micke Moreno, Luisa Zanolli Moreno, Júlia da Mata Góes Silva, Simone Koprowski Garcia, Dalton de Oliveira Fontes

**Affiliations:** 1 Technical Services Department, Agroceres Multimix, Rio Claro 13502-741, Brazil; felipe.alves@agroceres.com; 2 Artemis Ambiental, Pará de Minas 35661-009, Brazil; starosa@gmail.com; 3 Japfa Comfeed Vietnam, Vĩnh Phúc 02113, Vietnam; maucdutra@hotmail.com; 4 Faculty of Veterinary Medicine and Animal Science, University of São Paulo, São Paulo 05508-270, Brazilluisa.moreno@alumni.usp.br (L.Z.M.);; 5 Department of Animal Science, Veterinary School of the Federal University of Minas Gerais, Belo Horizonte 31270-901, Brazil; simonekg@vet.ufmg.br (S.K.G.); dalton@vet.ufmg.br (D.d.O.F.)

**Keywords:** pig farming, animal health, biosecurity, antimicrobial, additives, resistance

## Abstract

The use of antimicrobials in swine production is an issue that concerns the whole world due to their impact on animal and public health. This study aimed to verify the antimicrobial use in 29 commercial full-cycle farms in the midwestern region of the state of Minas Gerais, since this region is a hub of intensive pig farming in Brazil, as well as the possible correlations between the use of antimicrobials, biosecurity, and productivity. A total of 28 different drugs used for preventive purposes were described. On average, the herds used seven drugs, exposing the piglets for 116 days and totaling 434.17 mg of antimicrobials per kilogram of pig produced. Just eight active ingredients made up 77.5% of the total number of drugs used on the studied herds. Significant differences were found between the variables, biosecurity score and number of sows, antimicrobial amount and number of drugs, number of drugs and number of sows, and between productivity and biosecurity scores. The use of antimicrobials was considered excessive in the swine farms in the state of Minas Gerais compared to what was reported in Brazil and in other countries. Educational measures and better control should be proposed to reduce the preventive use of antimicrobials.

## 1. Introduction

Brazil is recognized worldwide for its intense swine production, being considered the fourth largest producer and exporter of pork [1]. However, to achieve such conditions, it is necessary to maintain strict biosecurity and food safety measures. In this case, one can mention epidemiological control and animal health protection, which are governed by the National Program for Suidae Health (PNSS) by the Ministry of Agriculture, Livestock and Supply (MAPA), following the international recommendations of the animal sanitary code of the World Organization for Animal Health (WOAH). As in other countries, Brazilian commercial pig farming is concentrated in regional hubs. Among the swine producing regions, the state of Minas Gerais stands out, having about five million pigs distributed in three expressive hubs: the Triângulo Mineiro/Alto Paranaíba, Zona da Mata Mineira, and the central region. 

The central region of the state has farms of different sizes, linked or not to the local cooperative systems and with strong participation in the supply of the capital, Belo Horizonte. Pig farming represents, together with poultry, a sector of great socioeconomic importance for the region. However, the intense farming and agro-industrial activity and the high density of swine per km^2^ require greater attention with epidemiological surveillance and more strict biosecurity measures at the farm level, both to prevent the introduction of new pathogens and for the control and containment of prevalent pathogens. In addition to biosecurity measures, the use of antimicrobials is also one of the practices used to prevent and contain diseases on these farms.

According to a survey conducted by the WOAH, a large part of the 35 reporting countries uses antimicrobials as growth promoters, and 57% of them do not have specific legislation for their use. In countries where there are regulations, there is a list of antimicrobials that have some restrictions for veterinary use [2]. In Brazil, the National Action Plan on Antimicrobial Resistance in Agriculture (PAN-BR AGRO) was created with the general objective to guarantee the capacity for treatment and prevention with effective drugs, used responsibly and with accessibility to all. Such initiatives comply with the WHO, FAO, and WOAH Global Action Plan on Antimicrobial Resistance, which consider the issue to be strategic for public health and global food security [3].

Van Boeckel et al. [4] describe that most countries do not collect or release data on the veterinary use of antimicrobials. In Brazil, the intensive production system and national regulations favor the continued use of antimicrobials in feed, while there is still little awareness about biosecurity, where only reproduction nucleus farms are required to follow legislation [5]. Previous studies have identified the use of large quantities of antimicrobials per kilogram of pork produced on Brazilian farms as a preventive measure, as well as pointed out deficiencies in the implementation of internal and external biosecurity measures [6,7]. The objective of this study was to verify the use of antimicrobials in full-cycle commercial farms in the midwestern region of the state of Minas Gerais, to determine possible correlations between the use of antimicrobials, biosecurity, and productivity.

## 2. Results

### 2.1. Size and Productivity of Swine Farms Evaluated

This study involved 29 commercial full-cycle farms, with averages of 549.36 sows per farm, ranging from 22 to 3208 sows. Productivity, represented by the number of terminated piglets per sow, per year, multiplied by the average weight of piglets terminated at slaughter, was, on average, 2832.90 kg pig/sow/year and ranged from 2074.27 to 3668.00 kg pig/sow/year. The average daily weight gain from birth to slaughter was 0.670 kg, ranging from 0.599 to 0.738 kg between farms. The average biosecurity score obtained for the studied herds was 587 points, ranging from 260 to 920 points (Table 1). 

### 2.2. Use of Antimicrobials

During the interviews, 28 antimicrobials were mentioned, whose forms of administration in the different stages of pig rearing are shown in Table 2. Only two forms of administration of antimicrobials were found in the herds: injectable (intramuscular) and oral (in-feed). Of the 13 reported classes, bambermycin (FLA) and streptogramin (VIRG) were only used in the growing and finishing phases, while hydroxyquinoline (HAL) was only used in the suckling and nursing phases. The other classes were used in all different stages of pig rearing. Some drugs like aminoglycosides (GEN), β-lactams (AMO and PEN), lincosamide (LIN), and quinolones (ENO) were used in both forms (injectable and oral) for suckling piglets.

**Figure 1:**
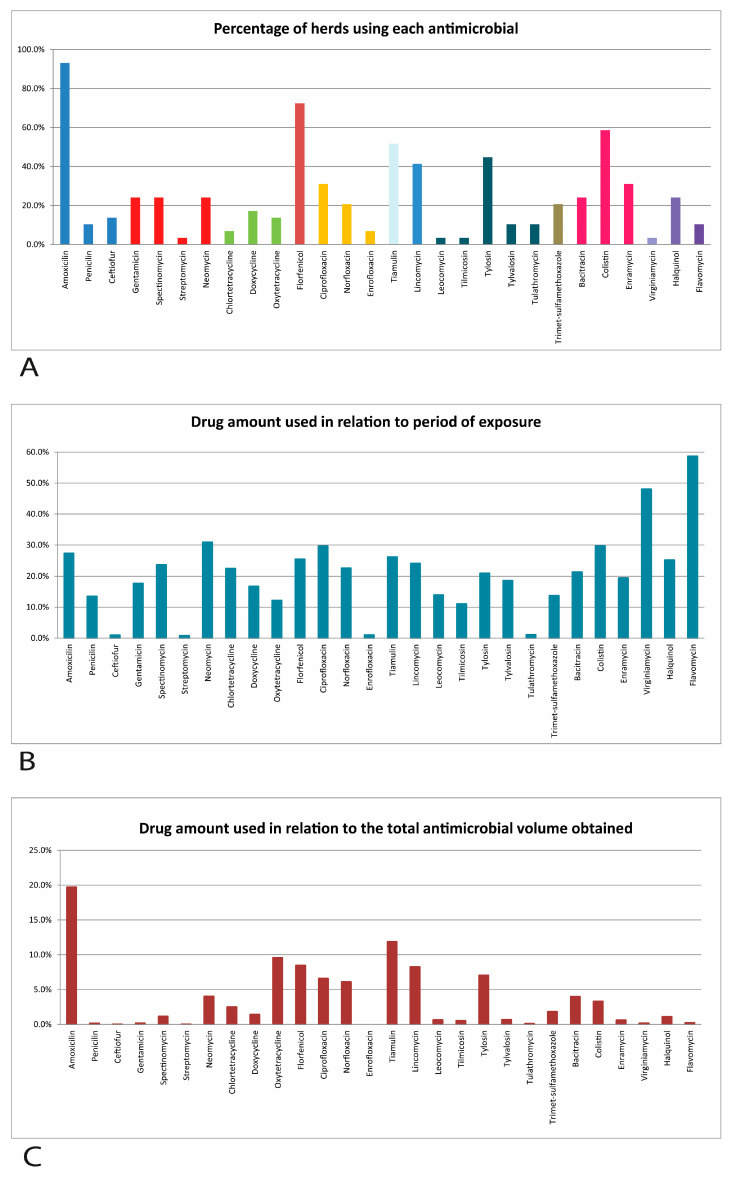
Percentage of herds using different antimicrobials (**A**) and proportion used in relation to the period of exposure (**B**) and in relation to the total quantity used (**C**) in the commercial full-cycle pig farms evaluated in the central region of the state of Minas Gerais.

According to the results shown in Table 1, the 29 farms used, on average, 434.17 mg of antimicrobials/kg of pig produced, ranging from 34.17 to 1145.96 mg/kg of pig produced. The average number of drugs used on each farm was 7.41, ranging from 3 to 13 different drugs per farm. As shown in Figure 1, the most frequently mentioned antimicrobials on the studied farms were amoxicillin (93.1%), florfenicol (72.4%), colistin (58.6%), tiamulin (51.7%), tylosin (44.8%), and lincomycin (41.4%). 

The animals were exposed to different antimicrobials for 116.55 days on average, ranging from 49 to 162 days (Table 1). In Table 3 and Figure 1, it can be observed that, among the antimicrobials that were most frequently mentioned by the farms, the animals were exposed to colistin (32.27 days), amoxicillin (30.74 days), and florfenicol (29.38 days). However, there was much variation between farms in the period of exposure to each antimicrobial.

The antimicrobials with the highest frequency of use in relation to the total were amoxicillin, tiamulin, oxytetracycline, florfenicol, lincomycin, tylosin, ciprofloxacin, and norfloxacin. Only these eight active ingredients made up 77.3% of the total quantity of antimicrobials used on the studied farms (Table 3).

The antimicrobials used in larger amounts were present in the growing phase, as shown in Table 4. In the farrowing phase, 15 drugs were used, totaling 1.35% of the total quantity (Table 2). Six of them made up 95.06% of the quantity used in this phase, with emphasis on neomycin, followed by amoxicillin, ciprofloxacin, and colistin. In the nursery phase, 19 antimicrobials were used, totaling 28.04% of the total quantity. Ten of them made up 94.26% of the quantity used in this phase, with emphasis on amoxicillin, followed by neomycin, colistin, tiamulin, and ciprofloxacin.

The use of antimicrobials in the growing and finishing (GF) phases was jointly recorded; subsequently, the data were separated by phase, according to the age at the beginning and end of exposure to each active ingredient. Thus, in the two phases (GF), 19 antimicrobials were used, totaling 922,823.98 mg of antimicrobials, or 70.61% of the total quantity. Ten of them made up 90.43% of the quantity used in these phases, with emphasis on amoxicillin, followed by tiamulin, florfenicol, oxytetracycline, lincomycin, and tylosin.

In the growing phase, 17 active ingredients were used, totaling 41.36% of the total amount. Ten of them made up 92.59% of the quantity used in this phase, with a small emphasis on tiamulin, whose value was very close to that of amoxicillin, florfenicol, and lincomycin, followed by norfloxacin. Finally, for the pigs in the finishing phase, 14 antimicrobials were used, or 29.25% of the total quantity. Eight of them made up 92.45% of the quantity used in this phase. Oxytetracycline, which had the highest percentage participation in the total quantity of antimicrobials used in this phase, was used in only two (02) of the 29 studied farms. Other highlights were amoxicillin, tylosin, and bacitracin (BMD/BZN).

All the farms studied declared using antimicrobials only for preventive purposes, mainly orally, in the feed. For many antimicrobials, the veterinary recommendation for this purpose and route of administration is in periods of 5 to 14 days. The choice of the drug depends on the disease or pathogenic agents in focus. Table 5 shows how the main antimicrobials, among those indicated in Table 4, were used in the nursery, growing, and finishing phases, considering the average data.

### 2.3. Relationships between the Studied Variables

**Figure 2:**
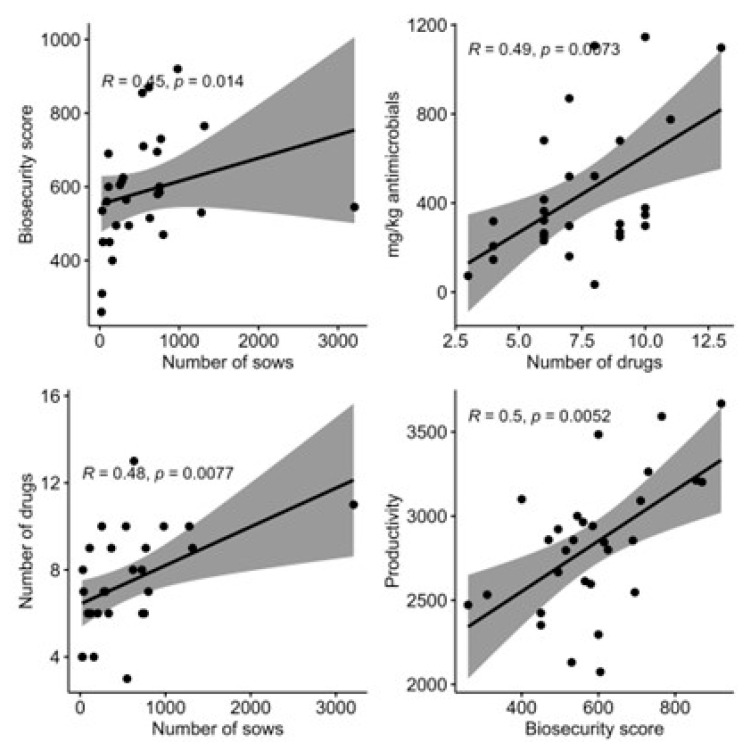
Graphs of significant correlations (*p* < 0.05) between the studied variables.

Table S1 presents the correlation matrix between the variables studied, graphically represented in Figure 2.

**Figure 3:**
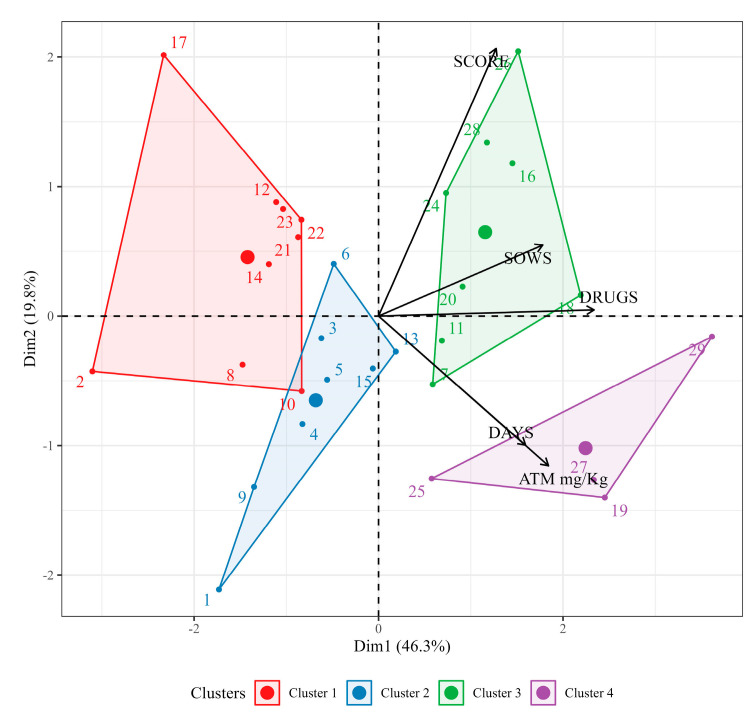
Formation of hierarchical groups (clusters) of the main components.

The principal component analysis (PCA) resulted in two dimensions explaining 66.10% of the data variance. Dimension 1 accounted for 46.34% and was basically explained by the quantity of antimicrobials used (33.72%), while dimension 2 accounted for 19.76% and was explained by the biosecurity score (61.87%). The formation of principal component groups (clusters), made with the HCPC method, resulted in four clusters, characterized in Table S2 and represented in Figure 3.

Cluster 1 differs from the others by the low number of antimicrobials (3 to 7 drugs per farm) and shorter period of exposure of the piglets to antimicrobials (49 to 90 days). Cluster 2 is of smaller farms, with fewer sows. Cluster 3 differs by having the highest biosecurity score, on average. Finally, Cluster 4 differs by having the highest quantity of antimicrobials/kg of live weight and the highest quantity of drugs (Table 6).

**Figure 4:**
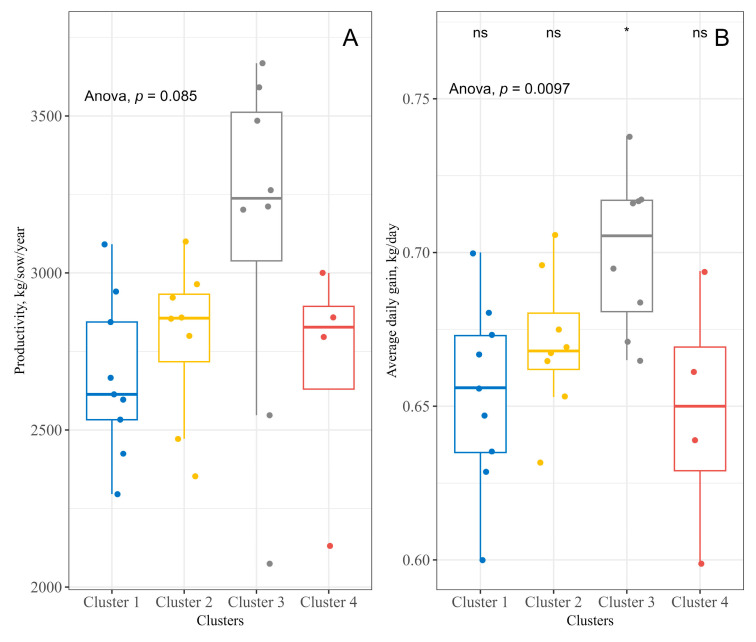
Difference in means between the clusters for the dependent variables productivity (**A**), in kg of swine produced, per sow, per year, and daily weight gain of the piglets from birth to slaughter (**B**).

According to Figure 4B, the characteristics that differ to form the groups affected the daily weight gain variable of the piglets, from birth to slaughter, whose average was significantly higher in Cluster 3 (*p* = 0.009). On the other hand, the productivity in Cluster 3 only tended to differ from the other groups (*p* = 0.085) (Figure 4A).

The results of the multiple linear regression analysis (stepwise regression), shown in Table S3, indicated biosecurity score as the variable that best explains daily weight gain and productivity, regardless of the other variables. Productivity explained 29.9% of the variability, with the biosecurity score increasing with productivity (*p* = 0.001). Daily weight gain explains 8.8% of the variability, and biosecurity only tends to relate to it (*p* = 0.065).

## 3. Discussion

Several studies have been carried out around the world to assess biosecurity in pig farms and establish a relationship with the use of antimicrobials and their effects on the health and productivity of herds [7,8,9,10,11,12]. Despite the practical advantages of farm biosecurity on animal health and welfare, farmers and animal health professionals in resource-limited regions may still be hesitant about its efficacy in substituting the use of preventive antimicrobial use [13]. 

There was great variation in the size of the 29 farms, from 22 to 3208 sows, and in productivity, from 2074.27 to 3668.00 kg of hog/sow/year, indicating possible differences in the adoption of breeding technologies, management, or sanitary status of the facilities between the farms. It is worth remembering that zootechnical results are multifactorial; therefore, some factors such as different installations, genetics, nutritional management, and health status could explain the variation in productivity between farms. Although surveying these factors was not the object of this study, a good indicator would be the preventive measures adopted by the farms—such as periodic serological monitoring and the use of vaccines, among other measures—as part of a biosecurity program. In fact, the analysis of correlations in this study indicated that farms with a higher number of sows have a higher biosecurity score and that farms with a higher biosecurity score are more productive.

It was observed that 21 of the 29 studied farms use antimicrobials preventively as a routine for suckling piglets in the farrowing pen, and 62% of them use more than one drug: a total of 15 active ingredients were administered in this phase, among them, five intramuscularly, six orally (in feed), and four administered in both ways (GEN, AMO, PEN, and LINCO). These results are similar to those described in 2016, in Brazilian herds [6], where 72% of the studied farms used antimicrobials preventively in the farrowing phase and half of them used more than one active ingredient, especially CEF, AMO, GEN, and LINCO. It was also found that seven drugs are being used preventively for suckling piglets in the farrowing pen (CEF, TUL, AMO, GEN, LIN, ESP, and BMD) [7]. These results portray a complex reality, since this use can affect the population of microorganisms, such as bacteria that naturally inhabit the gastrointestinal and respiratory tract, favoring the development of bacterial resistance. 

An important problem related to excessive antimicrobial use is that many farmers are not aware of the dangers of excessive antimicrobial use and its consequences, as well as it being favored by the lack of federal monitoring that does not have the structural capability to monitor all registered farms. The USA, China, and Brazil are the main global antibiotics users on an industrial scale [14]. Current evidence shows that more than 75% of medicated feed is used in pig farms, and it calls for urgent intervention [14].

Several antimicrobials described in the studied herds and cited by the owners and managers are classified by the WHO and OIE as “Highest Priority Critically Important Antimicrobials” [15,16]. Policies aiming at the prudent use of antibiotics do not recommend the use of these principles mentioned by farmers, such as aminopenicillins, tetracyclines, macrolides, quinolones, and amphenicols, for the prevention or treatment of pigs, given their association with AMR in humans [16]. 

Normative Instruction No. 45/2016 of MAPA [17] prohibits the use of colistin sulfate as a performance-enhancing additive in animal feed. However, more than 58% of the farms in this study use this drug, with a long period of exposure, although with a low proportion in relation to the total quantity of antimicrobials. Regulatory instructions do not prevent the therapeutic use of colistin, which remains permitted. The data obtained in this study indicate the use of colistin in a similar way to growth promoters, pointing to a possible inappropriate application of such a product. [18]. Callens et al. [18] presented a similar situation in Europe and reported that, even with antimicrobial growth promoters being banned since 2006, there is continuous use with justifications for treatment, control, or prevention of infectious diseases.

Drugs such as virginiamycin and flavomycin were used only in the growing and finishing phases and only in a few farms, in small quantities in relation to the total quantity of antimicrobials, but during a long period of exposure, since these were used as growth promoters. Previous reports described that less than 20% of state of Santa Catarina pig farmers knew how to differentiate the prophylactic use of antimicrobials from their use as growth promoters and that 29% could not say whether they used antimicrobials for this purpose in the feed [16].

Considering the antimicrobial use in each rearing phase (Table 4), the growing phase presented 41.33% of the amount used, which reveals the importance of reducing antimicrobials at this stage. The antimicrobials tiamulin, tylosin, and lincomycin, which were used in a large proportion at the time of the interviews, will have to be reassessed, as they are already prohibited as additives to improve animal performance in Brazil by IN1/2020 [17]. 

The antimicrobials amoxicillin and tiamulin follow dosages close to the usage indications of the main manufacturers found in the farms, 20mg/kg and 8.8 mg/kg, respectively. However, the indicated period of use is a maximum of 7 consecutive days for amoxicillin and 10 days for tiamulin. Table 5 shows the exposure of 13.25 to 22.5 days in the nursery phase, 15 to 18.29 days in the growing phase, and 15.25 days in the finishing phase for amoxicillin and exposure of 10 to 16.18 days in the nursery phase, 16.38 in the growing phase, and 16.25 days in the finishing phase, showing greater exposure than indicated. Florfenicol follows the same pattern, with dosages according to the main reported manufacturers (2 to 15 mg/kg, for 14 days) reaching 18.33 days of exposure in the growing season. 

The use of lincomycin (Table 5) ranges from underdosages in the nursery phase (1.1 mg/kg) to overdoses in the growing (15.51 mg/kg) and finishing phases (12.1 mg/kg), with exposure periods from 13.6 days in the nursery phase to 20 days in the rearing phase. This shows inappropriate use of the product, since the reported indication of the main manufacturers is the preventive use of 5 mg/kg with exposure of up to 14 days or curative use of 10 mg/kg and exposure of a maximum of 10 days, thus demonstrating excessive exposure to this antimicrobial, regardless of the rearing phase. This study contemplated the use of antimicrobials from birth to slaughter, which shows great variation in the period of exposure to the different drugs. Other studies have confirmed this variation and the prolonged use of antimicrobials, exceeding the prescription time [7].

In the present study, all interviewees from the 29 farms declared that they only use antimicrobials in all stages of pig production for preventive purposes. Studies point out the routine use of antimicrobials in pig farming, with different purposes, especially preventive, to reduce possible damages in a disease outbreak [8]. Producers claim antimicrobial use to be a “necessary evil” for disease prevention [16]. Authors also consider it a frequent and common practice in pig farming, but which represents an imminent risk in the selection of bacterial resistance [12]. 

As for the forms of use, there was a predominance of the oral route (in feed) in weaning pigs and the growing–finishing phases and the parenteral route (intramuscular) for suckling piglets in the farrowing phase. The effectiveness of antimicrobial use in feed can be questionable since sick animals can suffer from a lack of appetite [19]. The animal, even when sick, still ingests some amount of water, which favors the treatment of groups via drinking water, ensuring more precision and efficiency compared to the feed [20]. Like all technology, some precautions must be taken to ensure the process works, such as the palatability of the water after dilution, drug solubility, and accurate measurement of daily water consumption in the pen or batch [20].

The average consumption of antimicrobials was 434.17 mg/kg of pig produced, a quantity 17.5% higher than the 358 mg/kg of pig produced described in Brazil earlier [6]. This finding suggests that there is excessive use of antimicrobials in Brazilian pig farming and that the situation is not improving. In a similar survey [4], it was found that the global average consumption of antimicrobials is 172 mg/kg of pig produced, and Canada [21] reported the use of 150 mg/kg for the production species. The number of antimicrobials used did not correlate directly with biosecurity or productivity, as in other studies [7].

From the results found, it can be inferred that the quantity of antimicrobials is not directly related to the daily weight gain, but the higher the biosecurity scores, the greater the opportunity to remove antimicrobials. Perchance, producers or field technicians do not feel safe in reducing the use of antimicrobials, once adopted as a routine. The literature describes a negative association between the use of antimicrobials and biosecurity [9], indicating that farms where there is greater biosecurity have a lower frequency of antimicrobial treatment in different categories. Dhaka et al. [13], analyzing the literature, established that, besides biosecurity, several other farm management factors influence antimicrobial use in the herd. These factors include farm structure, animal health status, disease prevalence or risk of outbreaks, farmers’ socioeconomic and educational status, farmers’ and animal health professionals’ attitudes towards biosecurity and management practices, and regional or national stewardship policies.

The use of 28 drugs belonging to 13 antimicrobials classes was verified in all stages of piglet rearing. A previous study evaluating herds in several Brazilian states describes the use of 26 drugs, from 14 antimicrobial classes [6], which confirms the worrying picture in Brazilian swine production. The antimicrobials most frequently cited in interviews, in this study, were amoxicillin, florfenicol, colistin, tiamulin, tylosin, and lincomycin. In addition to the frequency with which they are mentioned, it was verified that only eight drugs corresponded to 77.5% of the total quantity of antimicrobials used, and they were amoxicillin (19.7%), tiamulin (11.85%), oxytetracycline (9.57%), florfenicol (8.44%), lincomycin (8.23%), tylosin (7.03%), ciprofloxacin (6.59%), and norfloxacin (6.10%). In addition, there was an accumulation of antimicrobials per farm, with an average of 7.45 different drugs, and the average period of exposure to these drugs was 116.5 days but with great variation between farms and results similar to those found in other publications [6].

In a study with independent pig farmers from a hub in Santa Catarina, it was described that antimicrobials could be easily acquired in agricultural stores or from supplies vendors (and can even be ordered via cell phone messages), either in powder form, to be added to the feed, or injectables, without the need for a veterinary prescription [16]. This demonstrates that, at that moment, farmers had free access to antimicrobials, which facilitates indiscriminate use in pig farming. Since June 2023, the Brazilian Government has determined new rules for the handling of antimicrobials to be added to animal feed and regulated this method of prophylactic use, requiring a prescription from a veterinarian, as well as new standards and rules for establishments that will prepare the medicated feed (SDA No. 798) [22]. It is expected that this new regulation will be able to reduce the preventive use of antimicrobials in feed and encourage the adoption of good production practices, biosecurity, and animal welfare in the country. 

## 4. Materials and Methods

### 4.1. Farms

In December 2020, the commercial pig farms in the midwestern region of Minas Gerais were identified, together with the Regional Coordination of Bom Despacho, Sectional Office of Pará de Minas, linked to the Instituto Mineiro de Agropecuária (IMA). As commercial farms, all those that sell pigs accompanied by the Animal Transit Guide were considered, regardless of the specialization or purpose of production and size of the farm. 

Pig breeding farms, insemination centers, piglet production units (PPUs), and growing/finishing units (RTUs) were excluded from this study. Thus, only commercial full-cycle (FC) farms were considered, which have the reproduction, gestation, farrowing, nursery, growing, and finishing sectors in the same breeding site. Therefore, a total of 29 commercial full-cycle farms were included in this study.

### 4.2. Data Collection on the Farms

Between January and March 2021, a single researcher carried out visits to the farms to collect data through personal interviews with the owner and/or herd manager. Data were collected following the classification proposed by the Association of Swine Veterinarian’s Production Animal Disease Risk Assessment Program (AASV-PADRAP) and as previously adapted using a form about biosecurity and a second form about health management [7]. Form I consisted of 120 questions on 16 aspects of biosecurity, involving the existence of a biosecurity program, characteristics and location of the farm, circulation of employees and visitors, isolation and quarantine, equipment, pest control, acquisition of supplements and semen, water and air quality, transport of animals and feed inputs, general maintenance and hygiene management and handling of dead animals, garbage, and waste. These data served as the basis for classifying the farms in terms of biosecurity or health risk, as each question was scored with zero (inadequate), five (requires adjustments), or ten points (adequate), considering the statements on the use or application of the practice in focus. 

Form II concentrated the responses on the use of antimicrobials, with the main drugs, dosages (daily and total), and application routes (feed, water, oral, or injectable solution) in each phase of pig rearing (suckling, nursing, growing, and finishing), characterized by age at the time of drug administration. Other data were the average age and average live weight at slaughter, from which the average daily weight gain (ADWG) from birth to slaughter was obtained. 

Productivity, given in kilograms of swine produced, per sow, per year, was obtained from zootechnical records consisting in the computerized program of the company Agriness S2 (Florianópolis, Brazil), used on most farms. On farms A1, A3, A5, A6, and B12, which have manual zootechnical records, productivity was estimated from the interviewee’s statement on the average number of terminated piglets sold per month, multiplied by the average live weight at slaughter. The result was multiplied by 12 months and divided by the number of sows on the farm.

### 4.3. Data Processing

Data were tabulated in Excel^®^ spreadsheets (Microsoft, 2020) for statistical analysis. Data from Form I were tabulated, and the scores for each response were added to rank each farm for biosecurity. The sum of the points could indicate six levels of health risk: extreme high risk (0 to 300 points), high risk (301 to 600), medium-high risk (601 to 900), medium risk (901 to 1000), low-medium risk (1001 to 1100), and low risk (1101 to 1200 points).

The proportion of each antimicrobial (in milligrams) used per kilogram of swine produced (mg/kg of swine) in each farm was calculated. For intramuscularly administered antimicrobials, the data, collected in milliliters of the commercial product per kilogram of body weight, were converted (according to product concentration) into mg of active ingredient/mL of product and then into mg/kg of live body weight. For antimicrobials administered in the feed, data were collected in grams (g) or in parts per million (ppm) of the drugs per ton (ton) of feed (g/ton or ppm/ton).

Calculations considered milligrams of antimicrobials per kilogram of pig produced in each herd and the period of use (mg/kg biomass), as previously described [7]. The following age categories were used for the calculation: suckling piglets (birth to an approximate weight of 6 kg—weaning), weaners (weaning to an approximate weight of 30 kg), fatteners (~30 kg to slaughter), and adult pigs. The kg animal at risk is the total weight of pigs for that age category (in kilograms).

The total quantity of antimicrobials used by the farms, in milligrams, was obtained by adding the quantities used in each farm in the different stages of piglet rearing. By dividing the total quantity of antimicrobials by the average live weight sold, the quantity of antimicrobials per kilogram of live weight, in mg/kg of swine, was obtained, as adapted previously [6].

### 4.4. Statistical Analysis

The data, tabulated and treated in Excel^®^ spreadsheets (Microsoft, 2020), were submitted to descriptive analysis, considering the averages and relative frequencies of the variables studied, by farm. First, the following variables were taken as independent: number of sows on the farm (MATRICES), biosecurity score (SCORE), amount of antimicrobials used in mg/kg of pig produced on the farm (ATM), number of drugs used on the farm (DRUGS), and duration of antimicrobial use, in days (DAYS); daily weight gain from birth to slaughter (DWG) and productivity in kg produced per sow per year (PROD) were also considered as dependent variables.

Spearman’s rank correlations were performed between all the variables to detect collinearity and how the variables were related, using the rcorr function of the Hmisc package [23]. Then, principal component analyses (PCAs) and hierarchical clustering of principal components (HCPC) were conducted using the PCA and HCPC functions of the FactoMineR package [24], with which four clusters were identified [25]. 

Subsequently, productivity and daily weight gain of each cluster were compared by ANOVA, followed by Tukey’s post hoc test. In addition, a stepwise regression approach was used to determine which independent variables best explained the behavior of the dependent variables using the function ols_step_forward_p from the olsrr package [26], using a cutoff value of 0.30 to retain the predictor variable in the model. Any collinearities between the predictors were verified using the Variance Inflation Criterion, using the vif function of the car package [27]. For these steps, the assumptions of normality and homogeneity of variance of the model were previously verified, using the Shapiro–Wilk and Levene tests, respectively, using the shapiro.test function from the stat package (4.1.0) [28] and the leveneTest of the car package [27]. The statistical procedures were performed in version R 4.1.0 [28]. Significance and trend levels were 0.05 and 0.10, respectively, for all analyses.

## 5. Conclusions

The main conclusion of this study is that the preventive use of antimicrobial remains a big concern in Brazilian swine production. The new regulation to reduce in-feed antimicrobial usage can be an important step towards reducing abusive use, as can the establishment of biosecurity programs in commercial pig farms with the participation of the government, veterinary organizations, and livestock industry stakeholders.

## Figures and Tables

**Table 1:** Number of sows, productivity, biosecurity classification, and use of antimicrobials in the commercial full-cycle swine farms evaluated in the central region of the state of Minas Gerais.

Farms	Sows (N)	Productivity (kg/sow/year)	DWG *	Biosecurity **		Use of Antimicrobials		
				Punctuation	Risk ***	Quantity	Medicated Time	Drugs
			(kg)			(mg/kg pig)	(days)	(N)
TO 1	22	2472.00	0.665	260	EHR	318.26	144	4
A3	29	2532.41	0.680	310	RHR	206.78	49	4
A5	32	2857.14	0.667	535	RHR	34.17	140	8
A6	40	2352.00	0.653	450	RHR	297.62	127	7
A10	90	2964.00	0.669	560	RHR	364.11	138	6
B1	111	3484.71	0.671	600	RHR	680.11	124	9
B2	124	2424.00	0.673	450	RHR	415.59	81	6
B13	160	3100.00	0.706	400	RHR	145.84	162	4
B5	207	2666.66	0.667	495	RHR	681.39	87	6
B9	334	2613.12	0.600	565	RHR	263.07	90	6
B10	366	2921.93	0.696	495	RHR	269.50	125	9
C2	631	2796.00	0.639	515	RHR	1097.51	126	13
C4	730	2596.04	0.635	580	RHR	264.80	90	6
C3	752	2295.85	0.629	600	RHR	249.93	90	6
C13	753	2941.03	0.647	585	RHR	230.41	80	6
C8	800	2858.45	0.694	470	RHR	870.03	123	7
C9	1280	2130.75	0.599	530	RHR	1145.96	131	10
C12	3208	3000.00	0.661	545	RHR	775.40	144	11
A11	110	2854.44	0.632	690	HR	321.80	127	6
B6	254	2074.27	0.684	605	HR	378.05	138	10
B8	278	2843.62	0.656	615	HR	160.83	84	7
B7	295	2799.46	0.675	625	HR	519.31	136	7
B11	537	3212.11	0.717	855	HR	346.84	144	10
B12	550	3091.00	0.700	710	HR	72.98	50	3
C5	620	3202.00	0.695	870	HR	1106.55	144	8
C7	724	2547.00	0.665	695	HR	521.12	137	8
C6	770	3263.29	0.717	730	HR	248.78	128	9
C11	1318	3592.00	0.738	765	HR	306.44	120	9
C10	981	3668.00	0.716	920	MHR	297.82	121	10
Total	16,106							
Average	549.36	2832.90	0.670	587.07	RHR	434.17	116.55	7.41

* Daily weight gain; ** biosecurity classification; *** EHR—extremely high risk; RHR—really high risk; HR—high risk; MHR—medium high risk.

**Table 2:** Classes, forms of administration of the antimicrobials, and stages of rearing in which they are used in the commercial full-cycle pig farms evaluated in the central region of the state of Minas Gerais.

Antimicrobial Classes	Drugs		Forms of Administration	
	Abbreviation	Name	Intramuscular	In Feed
Aminoglycosides	GEN	Gentamicin	FA	FA, NP
	ESP	Spectinomycin	-	FA, NP, GF
	EST	Streptomycin	FA	-
	NEO	Neomycin	-	FA, NP
β-Lactams	AMO	Amoxicillin	FA	FA, NP, GF
	PEN	Penicillin	FA	FA, NP
	CEF	Ceftiofur	FA	-
Phenicol	FFN	Florfenicol	-	NP, GF
Diterpenes	TIA	Tiamulin	-	NP, GF
Lincosamides	LIN	Lincomycin	FA	FA, NP, GF
Macrolides	LEO	Leucomycin	-	GF
	TILM	Tilmicosin	-	GF
	TIL	Tylosin	-	NP, GF
	TYLV	Tylvalosin	-	NP, GF
	TUL	Tulathromycin	FA, NP	-
Tetracyclines	CLO	Chlortetracycline	-	NP, GF
	DOX	Doxycycline	-	NP, GF
	OXY	Oxytetracycline	FA	NP, GF
Quinolones	CIPRO	Ciprofloxacin (2nd generation)	-	FA, NP, GF
	NOR	Norfloxacin (2nd generation)	-	FA, NP, GF
	ENO	Enrofloxacin (3rd generation)	FA	MT
Sulfonamides	STX	Trimethoprim–sulfamethoxazole	-	NP, GF
Bambermycin	FLA	Flavomycin	-	GF
Streptogramins	VIRG	Virginiamycin	-	GF
Hydroxyquinoline	HAL	Halquinol	-	FA, NP
Polypeptides	BMD/BZN	Bac. Methylene Disalicylate	-	GF
	COL	Colistin	-	FA, NP,
	ENRA	Enramycin	-	GF

FA—suckling piglets in the farrowing phase; NP—piglets in the nursing phase; GF—pigs in the growing and finishing phases.

**Table 3:** Period of exposure to antimicrobials in the 29 full-cycle farms evaluated in the central region of the state of Minas Gerais.

Class	Antimicrobials	Farms (N)	Exposure Period (Days)			% Pig Life Exposure
			Min.	Max.	Average	
Aminoglycosides	Gentamicin	7	1	59	20.43	17.67
	Spectinomycin	7	1	42	24.29	23.61
	Streptomycin	2	1	1	1.00	0.75
	Neomycin	7	11	45	30.86	30.84
β-Lactams	Amoxicillin	27	1	84	30.74	27.23
	Penicillin	3	1	30	16.67	13.45
	Ceftiofur	4	1	2	1.25	0.95
Phenicol	Florfenicol	21	14	55	29.38	25.39
Diterpene	Tiamulin	15	10	64	31.20	26.12
Lincosamide	Lincomycin	12	1	67	27.25	24.13
Macrolides	Leucomycin	1	20	20	20.00	13.89
	Tilmicosin	1	14	14	14.00	11.02
	Tylosin	12	14	46	25.33	20.92
	Tylvalosin	5	15	28	23.00	18.55
	Tulathromycin	3	1	2	1.33	1.06
Quinolones	Ciprofloxacin	10	15	45	30.60	29.60
	Norfloxacin	6	19	74	31.33	22.58
	Enrofloxacin	2	1	1	1.00	0.98
Tetracyclines	Chlortetracycline	3	18	30	23.33	22.46
	Doxycycline	5	10	35	20.40	16.64
	Oxytetracycline	4	1	21	14.25	12.12
Sulfonamides	Trimethoprim–sulfamethoxazole	6	10	18	13.67	13.67
Bambermycin	Flavomycin	2	80	100	90.00	58.64
Streptogramins	Virginiamycin	1	59	59	59.00	47.97
Hydroxyquinoline	Halquinol	7	7	64	36.29	25.12
Polypeptides	Bacitracin BMD/BZN	7	10	43	28.00	21.31
	Colistin	16	12	59	32.27	29.69
	Enramycin	9	11	29	23.67	19.41

**Table 4:** Drugs with the highest quantity of use, per rearing phase, in the commercial full-cycle pig farms evaluated in the central region of the state of Minas Gerais.

Antimicrobials	% of Antimicrobials Used Per Rearing Phases					
	All Phases	Farrowing	Nursery	Growing and Finishing	Growing	Finishing
Amoxicillin	19.70	19.88	31.25	15.11	13.26	17.72
Tiamulin	11.85	0	9.97	12.82	14.76	10.06
Oxytetracycline	9.57	–	–	11.55	5.27	20.44
Florfenicol	8.44	0	3.50	11.84	13.36	8.26
Lincomycin	8.23	–	–	10.06	13.46	5.26
Tylosin	7.03	0	–	8.59	5.97	14.58
Ciprofloxacin	6.59	11.70	8.33	5.86	7.47	3.58
Norfloxacin	6.10	3.56	6.43	6.02	10.28	0
Others All stages	22.70					
Neomycin		44.27	12.17	0	0	0
Colistin		10.45	11.28	0	0	0
Halquinol		5.20	3.68	0	0	0
Others Farrowing		4.94				
Trimethoprim–sulfamethoxazole		0	3.92	–	–	0
Doxycycline		0	3.73	–	3.75	0
Other Nursery			5.74			
Bacitracin BMD/BZN		0	0	5.65	–	12.55
Chlortetracycline		0	0	2.93	5.01	0
Other GF				9.57	7.41	7.55
Total (mg)	1,306,917.70	17,689.84	366,403.46	922,823.98	540,566.74	382,257.66
% of the total	100%	1.35%	28.04%	70.61%	41.36%	29.25%

0 = no use of the ATM in the phase; dash = ATM use in the phase in a proportion less than 3%. Others = penicillin, gentamicin, spectinomycin, streptomycin, flavomycin, virginiamycin, ceftiofur, leucomycin, tilmicosin, tylvalosin, tulathromycin, enramycin, and enrofloxacin (3rd ger).

**Table 5:** Exposure of the piglets at different ages and daily doses of antimicrobials used in higher quantities in the commercial full-cycle farms evaluated in the central region of the state of Minas Gerais.

Antimicrobial	Pulse	Nursery					Growing					Finishing				
		N	Age (d)		Period (d)	Dose/day (mg/kg)	N	Age (d)		Period (d)	Dose/day (mg/kg)	N	Age (d)		Period (d)	Dose/day (mg/kg)
			Start	End				Start	End				Start	End		
Amoxicillin	1st	24	30.29	43.24	13.25	17.66	7	66.43	84.71	18.29	15.36	4	120.75	136.00	15.25	15.03
	2nd	12	43.08	59.08	16.00	15.31	1	96.00	111.00	15.00	12.50	-	-	-	-	-
	3rd	2	48.00	70.50	22.50	21.38	-	-	-	-	-	-	-	-	-	-
Tiamulin	1st	11	46.45	62.64	16.18	11.09	8	78.00	94.38	16.38	10.29	4	112.00	128.25	16.25	8.88
	2nd	1	50.00	60.00	10.00	9.00	4	98.25	114.25	16.00	8.88	-	-	-	-	-
Oxytetracycline	1st	1	36.00	43.00	7.00	37.50	1	93.00	107.00	14.00	42.50	2	111.00	128.50	17.50	35.00
Florfenicol	1st	10	40.50	55.30	14.80	4.83	13	77.38	94.54	17.15	5.62	6	123.33	138.67	15.33	4.58
	2nd	2	50.00	65.00	15.00	5.25	3	98.67	117.00	18.33	4.83	0	-	-	-	-
Lincomycin	1st	5	33.40	47.00	13.60	2.75	7	73.14	90.29	17.14	7.49	3	110.33	128.67	18.33	12.10
	2nd	1	36.00	50.00	14.00	1.10	1	101.00	121.00	20.00	15.51	-	-	-	-	-
Tylosin	1st	1	21.00	36.00	15.00	12.50	4	80.25	96.25	16.00	5.63	9	111.78	132.33	20.56	5.49
Ciprofloxacin	1st	7	28.14	42.71	14.57	12.68	3	80.33	95.33	15.00	14.17	1	111.00	125.00	14.00	15.00
	2nd	4	50.00	69.00	19.00	11.56	1	101.00	121.00	20.00	15.00	-	-	-	-	-
Norfloxacin	1st	5	42.00	61.20	19.20	15.17	2	82.00	104.50	22.50	15.93	-	-	-	-	-
	2nd	1	29.00	36.00	7.00	15.00	1	108.00	133.00	25.00	19.60	-	-	-	-	-

**Table 6:** Characteristics of the clusters according to the mean ± standard deviation and minimum and maximum values of the studied variables.

Variables	Cluster 1 (9 Farms)	Cluster 2 (8 Farms)	Cluster 3 (8 Farms)	Cluster 4 (4 Farms)
SOWS	417.44 ± 284.49 (29–753)	139.38 ± 127.89 (22–366)	664.38 ± 384.52 (111–1318)	1479.75 ± 1184.51 (631–3208)
SCORE	545.56 ± 114.63 (310–710)	501.88 ± 134.51 (260–690)	755.00 ± 120.30 (600–920)	515.00 ± 32.40 (470–545)
ATM	282.86 ± 175.26 (72.98–681.39)	283.83 ± 144.56 (34.17–19.31)	485.71 ± 287.59 (248.78–1106.55)	972.22 ± 178.01 (775.40–1145.96)
DRUGS	5.56 ± 1.24 (3–7)	6.38 ± 1.77 (4–9)	9.12 ± 0.83 (8–10)	10.25 ± 2.50 (7–13)
DAYS	77.89 ± 16.53 (49–90)	137.38 ± 12.12 (125–162)	132.00 ± 9.96 (120–144)	131.00 ± 9.27 (123–144)

## Data Availability

The data that support the findings of this study are available from the corresponding author upon reasonable request.

## Supplementary Materials

The following supporting information can be downloaded at: https://www.mdpi.com/article/10.3390/antibiotics13050403/s1, Table S1: Matrix of Spearman’s correlation between the studied variables. Table S2: Contribution (%) of the variables in each dimension by principal component. Table S3: Multiple linear regression models applied to studied variables.
