# Supplementary material for: Antimicrobial Use in Pig Farms in the Midwestern Region of Minas Gerais, Brazil
Source: Antibiotics (Basel). 2024 Apr 28;13(5):403. doi: 10.3390/antibiotics13050403 (PMC11117247; doi:10.3390/antibiotics13050403)
Supplement: Supplementary file 1 [file antibiotics-13-00403-s001.zip › antibiotics-2952193-supplementary.pdf]

Table S1 - Matrix of Spearman's correlation between the studied variables.

		SOWS	SCORE	ATM	DRUG	DAYS	PROD
Biosecurity	SCORE	0.45 *					
Quantity of antimicrobials	ATM	0.24	0.01				
No. of ATM	BASES	0.48 *	0.35 $	0.49 *			
ATM exposure days	DAYS	-0.09	0.04	0.31 $	0.37 $		
Productivity	PROD	0.29	0.50 *	-0.10	0.23	0.14	
Daily Weight Gain	DWG	0.07	0.30	-0.11	0.15	0.16	0.63 *Note

ATM- antimicrobial, * $p < 0.05$ and $ $p < 0.10$

Obs.: Collinearity effect between Daily Weight Gain X Productivity.

Table S2 – Contribution (%) of the variables in each dimension by principal component.

	Number of dimensions / Main components				
	1	2	3	4	5
Eigenvalues	2,317	0.988	0.836	0.538	0.321
Percentage of Variance, %	46,344	19,758	16,714	10,754	6,430
Accumulated variance, %	46,344	66.103	82,816	93,570	100,000
Variables					
SOWS	19.57	4.35	34.24	38.30	3.53
SCORE	10.04	61.87	8.92	9.28	9.90
ATM	20.97	19.34	10.50	35.68	13.51
DRUGS	33.72	0.03	0.59	1.09	64.57
DAYS	15.70	14.41	45.75	15.65	8.48

Table S3 - Multiple linear regression models.

**RESULT**	**PREDICTOR**	**ESTIMATE**	**STANDARD ERROR**	**P-VALUE**
PRODUCTIVITY				
Adjusted R^2 = 0.2994	intercept	1,951,388	252,732	<0.001
P = 0.001	SCORE	1.502	0.417	0.001
DAILY WEIGHT GAIN				
Adjusted R^2 = 0.0884	intercept	0.625	0.024	<0.001
P = 0.065	SCORE	0.000077	0.000040	0.065
